# The Extension of the Pension Fund

**Published:** 1890-01-25

**Authors:** 


### The Hospital

A WEEKLY JOURNAL OF

**Science, Medicine, Nursing, and Philanthropy.**

*For Hospitals, Asylums, Medical Practitioners, Students, Nurses, Families, Parishes, Congregations, and the Charitable Public.*

VOL. VII. No. 174.] [JANUARY 25, 1890.

### THE EXTENSION OF THE PENSION FUND.

THE National Pension Fund for Nurses has been firmly established on a permanent basis, and the consideration of its extension and improvement in detail are now the chief care and anxieties of the Council. The great cause for satisfaction is that the fund has been instituted without the payment of any preliminary expenses by the beneficiaries. To enable the Council to help nurses to the utmost, in the judgment of the actuary, Mr. George King, steps ought now to be taken for raising the amount of the Donation Bonus Fund to forty thousand pounds, or, better still, to fifty thousand, if the latter be found practicable. That, of course, would be a free gift to self-reliant nurses; and Mr. King is of opinion that with some such amount as this accumulating at compound interest until the pensions begin to fall due, every nurse who may join the fund ought to secure a minimum pension of ten shillings a week for life.

It is to be borne in mind that the figures of the Donation Bonus Fund are net figures, and represent a benefit to nurses of exactly the amount stated, without the deduction of a single penny for advertisements, printing, postage, or other usual expenses of collection. When it is considered that twenty or twenty-five per cent. is by no means an uncommon deduction from gross totals for cost of collection, the full significance of the extinction of this important item of expense is at once apparent. The Council of the Pension Fund have accepted, *con amore*, the suggestion of the actuary, and have resolved that the Donation Bonus Fund shall be raised to at least forty thousand pounds forthwith. THE HOSPITAL will undertake, for its part, the duty, not only of giving such stimulus to contributors as may be possible, but also the publication from week to week of a list of such benevolent persons as shall have collectively subscribed one thousand pounds, until the completion of at least £40,000.

We think we may, in passing, justly congratulate both hospitals and nurses on the extraordinary success of the Donation Bonus Fund. The forty thousand pounds named as so important a desideratum by the actuary is well within sight, and will no doubt be fully completed in the course of the next few weeks. With this amount as a constant source of revenue, the minds of all connected with the Pension Fund, either as responsible managers or as beneficiaries, are permanently freed from every cause of anxiety. But the fund having reached this stage of assured permanence, and being able to offer to nurses such great and undoubted benefits, it seems desirable that hospital managers in largely increased numbers shall consider how they may best avail themselves of its existence for the advantage of their nurses. We do not need to repeat, for the hundredth time, that nurses as a class are women of rare worth. They do work requiring the highest measure of intelligence and goodness for little more than the wages of domestic servants. Managers may not be able to increase their wages, but they can hardly be held blameless if they take no steps to help their nurses to secure some portion of the benefits of the Donation Bonus Fund. We are almost ashamed to appeal to a motive which is not particularly worthy in this connection. But managers cannot rid themselves of responsibility for nurses who, after devoting much time to their service, find themselves in poverty as age advances. The National Pension Fund, with its accumulated donation bonuses, offers to all committees of hospitals an excellent method of satisfying their consciences, and getting rid at once and for ever of all uneasy responsibility for sick and aged nurses. If managers would help their nurses when young to join the fund they would do as important a deed of benevolence as when they enlarge their buildings for the reception of additional patients, and they would at the same time secure for themselves that pleasant sense of inward satisfaction which invariably results from duty acknowledged and done. All hospitals we consider ought to feel bound to help their nurses to provide for sickness and old age by assisting their payments to the Pension Fund. But the managers of those hospitals that are at the same time training schools for nurses have undoubtedly a deeper obligation in this matter than others. It is well known that the private nursing institutions attached to the nurse training schools are at least moderately profitable. The arrangement existing between the managers of those institutions and the nurses they employ is, so far as we know, quite satisfactory and advantageous to both parties. But inasmuch as the profits made are due to the work and devotion of the nurses, it seems entirely right and reasonable that in addition to their bare wages a little of the profit also should be a part of their reward. In no way can such profit be so well spent on behalf of nurses as in helping them to pay their premiums to the Pension Fund. No manager of such a private nursing institution will consider we make anything but a modest request when we say that nurses should be assisted to the extent of half their premiums.

It is plain that the National Pension Fund with its bonus endowments is capable of rendering inestimable service to nurses. It is equally evident that it is the only fund in existence of its kind. The smaller pension schemes connected with different hospitals are for the exclusive use of persons connected with those hospitals. Meritorious as those schemes may be, and undoubtedly are, they cannot hope to offer even to their own members the benefits which the Pension Fund, by virtue of its ever-increasing bonus endowment, can confer upon them. In any case a large central organisation can always be worked at less expense and to greater advantage than a number of scattered small organisations of the same kind. The multiplication of centres, as of heads in an army, inevitably means additional cost to the commissariat. In view of these economic certainties, managers of small Funds will do well to take advantage of the present opportunity of affiliating themselves to what now may be considered the Parent Fund. One result of such affiliation would be an immediate increase of the area of supply to the central organisation. At the same time the managers of local funds would free themselves of all responsibility, whilst adding greatly to the prospective benefits of their nurses. Nurses themselves are inexperienced in business, and, therefore, sometimes timid. But necessity often makes cowards brave as well as fools wise; and they should think of the years that may be in store for them when the power of work shall be past. Even to the frivolous the prospect of falling into poverty after living in comfort and respect brings sober and not happy thoughts; whilst to the self-respecting and the educated the outlook is not to be endured. All these melancholy forecasts are swept away by the establishment of the National Pension Fund. Every nurse who has even a little foresight and self-denial may look forward to an old age of repose and comfort. The thing to bear in mind is that the Pension Fund can only give its benefits to those who join it. Its mere existence will help nobody. Both hospital managers and nurses owe it to themselves, as practical people, to consider all these things from a practical point of view, and, having considered, to take action in their own interests promptly.

